# Primary Malignant Melanoma of the Maxillary Sinus Initially Misdiagnosed as Large Cell Lymphoma: A Cytological Pitfall

**DOI:** 10.7759/cureus.92111

**Published:** 2025-09-12

**Authors:** Akemi Kobayashi, Akihiro Shioya, Toshie Terauchi, Yoshiiku Okanemasa, Sohsuke Yamada

**Affiliations:** 1 Department of Pathology, Kanazawa Medical University Hospital, Ishikawa, JPN; 2 Department of Pathology and Laboratory Medicine, Kanazawa Medical University, Ishikawa, JPN

**Keywords:** amelanotic malignant melanoma, diagnostic pitfall, fine-needle aspiration cytology, malignant melanoma, maxillary sinus

## Abstract

We report a case of malignant melanoma (MM) originating in the maxillary sinus in an 84-year-old man, who was initially misdiagnosed with large-cell lymphoma based on fine-needle aspiration cytology (FNAC). The cytological specimens showed a clear background with loosely cohesive, monotonous, and large atypical cells exhibiting high nuclear-to-cytoplasmic ratios and irregular nuclear contours without recognizable melanin pigment at the time of evaluation. These features led to the preliminary interpretation of malignant lymphoma. A biopsy specimen obtained from the nasal cavity revealed nests of atypical cells with enlarged, irregular nuclei, and prominent nucleoli. A few tumor cells contained brown granules, suggesting melanin pigmentation. Immunohistochemistry revealed tumor cells positive for HMB-45, melan-A, and SOX10, confirming the diagnosis of MM. A retrospective review of the cytological specimens revealed scattered pigmented tumor cells and intranuclear cytoplasmic pseudo-inclusions (Apitz bodies). This case illustrates a diagnostic pitfall in which amelanotic MM cytologically mimics large-cell lymphoma, particularly in the sinonasal region. Even in the absence of overt pigmentation, careful attention to subtle cytological features, such as Apitz bodies and scattered pigmented tumor cells, is essential, and MM should be considered in the differential cytological diagnosis of large-cell neoplasms.

## Introduction

Malignant melanoma (MM) arising in the head and neck region accounts for approximately 20% of all MMs [1,2]. Among these, MMs originating in the nasal cavity and paranasal sinuses are relatively rare, comprising only 8-10% of head and neck MMs [3]. Consequently, diagnostic opportunities are limited. The presence of melanin granules within tumor cells is an important finding in the cytological and pathological diagnosis of MM. Identification of such granules often raises suspicion of MM; however, amelanotic variants in which melanin deposition is minimal or absent also exist [4,5]. MM exhibits a wide variety of cellular morphologies, including epithelioid, spindle, and plasmacytoid forms. In cases where melanin granules are sparse, histological diagnosis can be challenging, with a broad differential diagnosis that includes undifferentiated carcinoma, sarcoma, and non-Hodgkin lymphoma [5-7]. Likewise, cytological diagnosis, which is also based on morphology, may be difficult owing to variable cytological appearances [8]. Herein, we report a rare case of MM originating in the maxillary sinus that was initially misinterpreted as a malignant lymphoma on fine-needle aspiration cytology (FNAC). However, such lesions should more appropriately be considered within the broader category of round cell tumors, given the many differentials that cannot be excluded without histopathology and immunohistochemistry. In this case, we discuss the diagnostic pitfalls of cytological evaluation of MM.

## Case presentation

An 84-year-old man presented with a swelling in the right cheek. His medical history included a radical prostatectomy for prostate cancer 15 years before presentation. No cutaneous lesions were noted previously. He was initially treated for apical periodontitis of the right maxillary premolars. However, symptoms did not improve, raising the suspicion of a neoplastic process. The complete blood count revealed no significant abnormalities, except for a mild decrease in red cell parameters. Serum lactate dehydrogenase (LDH) levels were elevated at 608 U/L, while soluble interleukin-2 receptor (sIL-2R) levels were within normal limits (251 U/mL) (Table 1).

**Table 1:** Laboratory tests. Laboratory tests revealed no abnormalities in the complete blood count. Serum lactate dehydrogenase levels were elevated, while sIL-2R levels were within normal limits. AFP, alpha-fetoprotein; Alb, albumin; ALT, alanine aminotransferase; AST, aspartate aminotransferase; BUN, blood urea nitrogen; CA19-9, carbohydrate antigen 19-9; CEA, carcinoembryonic antigen; CK, creatine kinase; CRP, C-reactive protein; D-bil, direct bilirubin; eGFRcreat, estimated glomerular filtration rate (calculated using creatinine); GGT, γ-glutamyl transferase; Hb, hemoglobin; Hct, hematocrit; LDH, Lactate dehydrogenase; MCH, mean corpuscular hemoglobin; MCHC, mean corpuscular hemoglobin concentration; MCV, mean corpuscular volume; PLT, platelet count; RBC, red blood cell count; SCC, squamous cell carcinoma antigen; sIL-2R, soluble interleukin-2 receptor; T-bil, total bilirubin; WBC, white blood cell count

Laboratory Findings	Units	Results	Reference Ranges
RBC	×10^6^/µL	3.8	4.35-5.55
Hb	g/dL	13.2	13.7-16.8
Hct	%	39.7	40.7-50.1
MCV	fL	103.5	83.6-98.2
MCH	pg	34.5	27.5-33.2
MCHC	g/dL	33.3	31.7-35.3
WBC	×10^3^/µL	6.4	3.3-8.6
Neutrophils (count)	×10^3^/µL	3.3	N/A
Lymphocytes (count)	×10^3^/µL	2.5	N/A
Neutrophils	%	51.1	40.0-77.0
Lymphocytes	%	38.2	16.0-44.0
Monocytes	%	6.2	4.0-9.0
Eosinophils	%	0.7	1.0-7.0
Basophils	%	0.3	0.0-1.0
Large unstained cells	%	3.5	1.0-4.0
PLT	×10^3^/µL	229.0	158.0-348.0
CRP	mg/dL	<0.02	0.0-0.14
Sodium	mmol/L	141.0	138.0-145.0
Potassium	mmol/L	3.8	3.6-4.8
Chloride	mmol/L	106.0	101.0-108.0
Calcium	mg/dL	9.1	8.8-10.1
Magnesium	mg/dL	1.9	1.8-2.4
BUN	mg/dL	16.0	8.0-20.0
Creatinine	mg/dL	0.9	0.65-1.07
eGFRcreat	mL/min/1.73m^2^	61.0	N/A
Serum iron	µg/dL	167.0	40.0-188.0
Total protein	g/dL	6.8	6.6-8.1
Alb	g/dL	3.8	4.1-5.1
LDH	U/L	608.0	124-222
T-bil	mg/dL	0.4	0.4-1.5
D-bil	mg/dL	0.1	0.0-0.4
AST	U/L	28.0	13.0-30.0
ALT	U/L	19.0	10.0-42.0
GGT	U/L	29.0	13.0-64.0
CK	U/L	64.0	59.0-248.0
Amylase	U/L	144.0	44.0-132.0
CEA	ng/mL	3.8	<5.0
AFP	ng/mL	3.1	<8.5
CA19-9	U/mL	<0.5	<37.0
SCC	ng/mL	0.6	<2.0
PIVKA-II (high-sensitivity)	mAU/mL	20.0	<40.0
sIL-2R	U/mL	251.0	157.0-474.0

**Figure 1:**
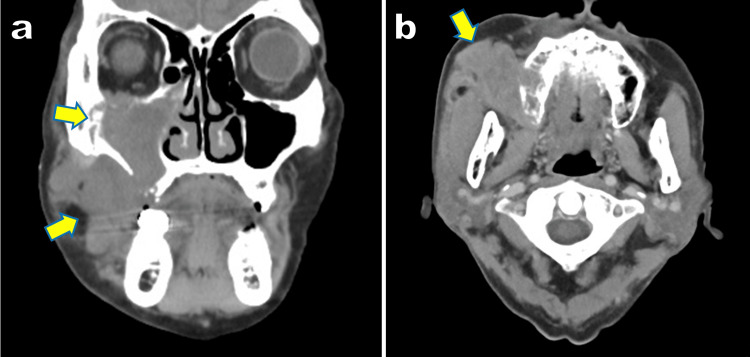
CT images. (a) Coronal section. (b) Axial section. A soft-tissue mass extending from the right maxillary sinus to the subcutaneous region of the right cheek and orbital floor. Destruction of the sinus wall and alveolar bone was also observed. The yellow arrows indicate the position of the soft-tissue mass.

Head and neck computed tomography (CT) revealed a soft tissue mass filling the right maxillary sinus, destroying the sinus wall and alveolar bone, and extending into the subcutaneous tissue, musculature of the cheek, and the orbital floor (Figure 1). The boundaries of the overlying skin were preserved. Positron emission tomography-computed tomography (PET-CT) demonstrated abnormal uptake in the right submandibular lymph nodes and segment 5 of the liver, suggesting metastasis.

**Figure 2:**
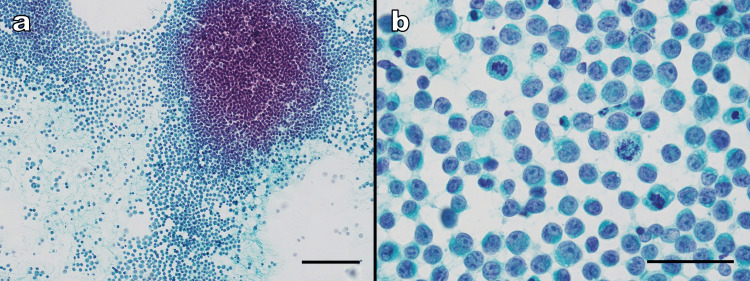
Fine-needle aspiration cytology (Papanicolaou stain). (a) Low-power view (×100; scale bar: 200 μm). The background is clear. Poorly cohesive atypical cells form dense clusters resembling lymphoid follicles. As one moves away from the clusters, the discohesive nature of the tumor cells becomes more evident, and they appear scattered and isolated. (b) High-power view (×600; scale bar: 50 μm). The atypical cells are medium to large, round to oval in shape, with a high nuclear-to-cytoplasmic ratio, nuclear irregularities, and a monotonous appearance. Some cells show eccentric nuclei, and mitotic figures are occasionally observed.

FNAC of the skin surface of the cheek mass was performed. Cytologically, the background was clear. Poorly cohesive atypical cells were densely clustered, mimicking lymphoid follicles. In areas further away from these clusters, the lack of cohesion became more apparent, with the tumor cells dispersed singly in a scattered pattern (Figure 2a). The atypical cells were medium to large in size, round to oval, with a high nuclear-to-cytoplasmic (N/C) ratio, nuclear irregularities, and monotonous chromatin features. Nuclear indentations, grooves, uneven chromatin distribution, and prominent nucleoli were also observed. Some cells exhibited eccentric nuclei, and mitotic figures were readily observed (Figure 2b). FNAC smears were stained with Papanicolaou stain. May-Grünwald-Giemsa staining was not performed. Based on cytological findings, the lesion was diagnosed as malignant, and malignant lymphoma was initially suspected.

**Figure 3:**
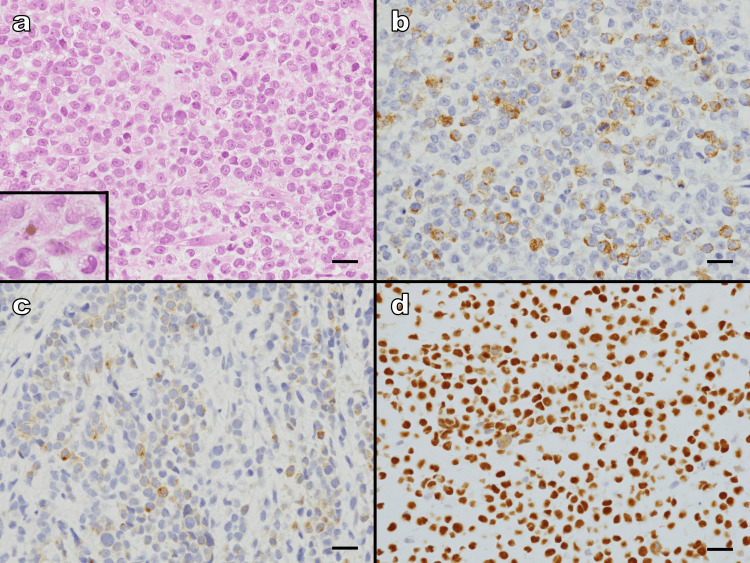
Histological examination. a) Hematoxylin and eosin staining shows poorly cohesive atypical cells with enlarged, irregular nuclei, prominent nucleoli, and a high nuclear-to-cytoplasmic ratio proliferating in a nest-like pattern. A few tumor cells contain brown granules suggestive of melanin pigment (inset). (b–d) Immunohistochemistry for melanoma markers shows positivity for (b) HMB-45, (c) melan-A, and (d) SOX10. (All panels: ×400; scale bars: 20 μm.)

Following cytological diagnosis, a biopsy was performed on the side of the nasal cavity of the right maxillary mass. Histologically, the tumor consisted of poorly cohesive atypical cells with enlarged, irregular nuclei, prominent nucleoli, and a high N/C ratio arranged in a nest-like pattern (Figure 3a). Careful examination revealed that a few tumor cells contained brown granules suggestive of melanin. Immunohistochemically, the tumor cells were positive for HMB-45, melan-A, and SOX10 (Figure 3), and negative for AE1/AE3, CAM5.2, S-100, LCA, CD3, CD20, CD34, desmin, and α-SMA. This immunoprofile effectively excluded carcinoma (AE1/AE3, CAM5.2), lymphoma (LCA, CD3, CD20), and sarcoma (CD34, desmin, α-SMA), leading to a definitive diagnosis of MM, rather than malignant lymphoma as initially suspected by cytology.

Histologically and immunohistochemically, the tumor was diagnosed as MM. Based on the clinical and imaging findings, including submandibular lymph node involvement and distant liver metastasis, the tumor was classified as stage IV. The primary tumor was technically resectable; however, surgery was not performed because distant metastasis was already present and radical treatment was not considered appropriate given the patient’s age. Combination therapy with nivolumab and ipilimumab was also avoided due to the patient’s age and the high risk of severe immune-related adverse events. The patient therefore received nivolumab monotherapy. After eight cycles, treatment was discontinued because of a left cerebral hemorrhage. The patient was transferred to another facility, and further follow-up was not possible.

## Discussion

MM presents a wide spectrum of cytological features, including epithelioid, spindle-shaped, and pleomorphic cells. These tumor cells are often discohesive and appear singly scattered, showing marked variations in size, abundant cytoplasm, large round nuclei, and prominent nucleoli [7,9,10]. Melanin pigment is typically yellow-brown on Papanicolaou staining and dark brown-black on May-Grünwald-Giemsa staining [7,9]. Intracytoplasmic or intranuclear pseudo-inclusions (Apitz bodies) can also be observed [7-10]. The background is usually hemorrhagic, and necrosis or melanin-laden macrophages may be present in some cases. However, accurate cytological diagnosis is difficult in the absence of these classic features [7,10].

In our case, the cytological background was clear, and the tumor cells were monotonous, round to oval in shape, large, and appeared as isolated discohesive cells. These features differed significantly from the typical cytological presentation of MM. Although cytology suggested malignancy, malignant lymphoma was initially suspected. This misinterpretation likely stems from the cytological similarity to large-cell lymphoma and the failure to recognize melanin pigments during the initial evaluation. Although amelanotic MM is relatively rare, accounting for approximately 13.2% of all MMs [11], some reports have shown that melanin pigments are not cytologically visible in over 50% of cases [7,9]. Therefore, even when melanin is sparse or absent, and the cytological pattern resembles that of large-cell lymphoma, MM should remain a diagnostic consideration.

Few cytological studies have specifically focused on primary MM in the sinonasal region. Most reports describe the cytology of cutaneous MM or its metastatic lesions [7-10]. Malignant lymphoma accounts for approximately 11% of all sinonasal malignancies [12,13], making it an important differential diagnosis. Common types include diffuse large B-cell lymphoma and extranodal NK/T-cell lymphoma, both of which typically present as monotonous populations of medium-to-large atypical lymphoid cells. In a study by Young et al. [14], among 283 cases of metastatic MM, 13 (4.6%) were misdiagnosed as non-Hodgkin’s lymphoma on cytology, highlighting a known diagnostic pitfall. The present case falls within this category.

**Figure 4:**
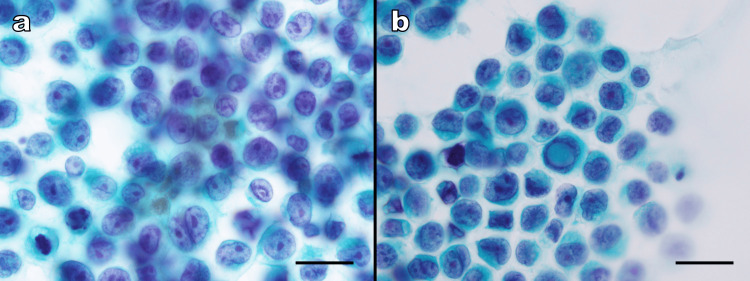
Cytological reevaluation of the present case (Papanicolaou stain). (a) A small area of melanin pigment deposition is observed. (b) A tumor cell with an intranuclear cytoplasmic pseudo-inclusion is identified. These findings, if noted at the cytological stage, could have provided strong motivation to search for melanoma markers during subsequent histological diagnosis. (Both images: ×1000; scale bars: 20 μm.)

Radhika et al. reported that nuclear pseudo-inclusions (Apitz bodies) were observed in 73% of MM cases, ranking third in frequency after pigmented macrophages (83%) and visible melanin pigments (77%). These features were not reported in the cytology of malignant lymphomas in the reviewed literature [6,12-14]. Thus, the presence of melanin pigments and nuclear pseudo-inclusions may serve as useful cytological markers to distinguish MM from malignant lymphoma. In retrospect, our case demonstrated sparse melanin granules and nuclear pseudo-inclusions upon reevaluation (Figure 4). Careful observation of these features during cytological assessment may provide important diagnostic clues and prompt further immunocytochemical investigation.

Immunohistochemistry is highly effective in the diagnosis of MM. SOX10, HMB-45, and melan-A are typically positive, and S-100 is often positive but may be negative, as in our case [10,11]. A broad range of tumors should be considered in the differential diagnosis [11-14]. For malignant lymphoma, immunomarkers help distinguish subtypes: LCA (CD45) as a pan-leukocyte marker, CD3 for T-cell lymphoma, CD20 for B-cell lymphoma, and CD56 for NK/T-cell lymphoma; EBER (Epstein-Barr virus-encoded RNA) in situ hybridization is also recommended for NK/T-cell lymphoma [6,7,9]. For carcinoma, including undifferentiated carcinoma, keratin markers such as AE1/AE3 and CAM5.2 are recommended, and p63 can support squamous differentiation [6,11]. For sarcomas, it is advisable to first examine basic markers related to vascular, neural, and muscular differentiation, such as CD34, S-100, desmin, and α-SMA [5,6,11-14]. In addition, olfactory neuroblastoma is an important differential diagnosis in this region; it should be considered when neuron-specific enolase, synaptophysin, and chromogranin are positive, and S-100 shows a sustentacular cell pattern [6].

The clinical presentation and cytological features of sinonasal tumors are often non-specific and may mimic other round cell neoplasms or even invasive fungal sinusitis, making histopathology with immunohistochemistry essential for the final diagnosis. This case also illustrates that rendering a definitive diagnosis of lymphoma on cytology alone in this location represented a diagnostic pitfall, underlining the need for biopsy confirmation or ancillary studies. Nevertheless, cytology remains a valuable and minimally invasive tool for the initial assessment. Careful attention to subtle morphological features, such as melanin pigment and nuclear pseudo-inclusions, can raise the suspicion of MM and provide a rationale for immunohistochemical evaluation in subsequent tissue biopsies. In this way, cytology serves not as a stand-alone diagnostic method but also as an important gateway that may help reduce diagnostic turnaround time and allow for earlier clinical management.

## Conclusions

In summary, we encountered a case of primary MM of the maxillary sinus that was cytologically misdiagnosed as malignant lymphoma. Even in cases with only sparse melanin pigments and cytological features mimicking lymphoma, MM should be considered in the differential diagnosis. Recognizing cytological clues, such as melanin deposition and nuclear pseudo-inclusions, is essential for the timely and accurate diagnosis of sinonasal MM.
